# Enhanced RX-Based Hyperspectral Anomaly Detection Using Laplacian-Regularized PCA

**DOI:** 10.3390/jimaging12070303

**Published:** 2026-07-06

**Authors:** Fatma Küçük

**Affiliations:** Department of Software Engineering, Ankara Yıldırım Beyazıt University, Ankara 06010, Turkey; fatmakucuk@aybu.edu.tr

**Keywords:** hyperspectral imaging, anomaly detection, Laplacian matrix, PCA

## Abstract

Hyperspectral anomaly detection application is essential in numerous different remote sensing applications, where the detection of rare and unknown targets in complex scenes is needed. The proposed anomaly detection method in this study is called L-PCAD (Laplacian PCA-based anomaly detection), which plans to combine a Linearized Alternating Direction method (LADM)-based subspace recovery algorithm with a modified RX detector to improve detection accuracy and stability. It starts with an LADM-based approach as a preprocessing stage to get a matrix with rich information relating to anomalies. The resulting low-rank background matrix is subsequently utilized as a guide for the anomaly detection process. In order to enhance the RX detector, the covariance estimation is reformulated using a graph Laplacian constructed from the low-rank background matrix. Instead of directly using the empirical covariance matrix, a normalized Laplacian is computed and subsequently transformed via principal component analysis (PCA) to obtain a stable diagonal representation. This PCA-regularized Laplacian replaces the conventional covariance matrix in the RX formulation while preserving the local spatial structure. The extensive testing of different hyperspectral datasets shows that the proposed approach provides better overall results for detection performance as compared to other hyperspectral anomaly detectors that are the state of the art.

## 1. Introduction

A hyperspectral image (HSI) is information that gives a set of 3D image data that encompasses the spatial and spectral characteristics of features as demonstrated in the earlier works of [1,2]. Hyperspectral remote sensing integrates these spatial data with spectral data with hundreds of adjacent spectral bands. This technology allows distinguishing various materials and such discrimination is proven by [3,4,5]. Through the integration of these spectral and spatial datasets, the technology provides unique capabilities for material identification and classification, even when operating within the visible spectrum. These capabilities enable users to gain awareness and extract detailed information about objects and environments. As a result, HSI technology has found wide-ranging applications in fields such as target detection in defense and surveillance [6,7], geological exploration and rare mineral detection [8,9], precision agriculture [10,11], food and pharmaceutical quality control [12,13], classification [14,15,16,17] and civilian search and rescue operations [18,19]. Among the diverse research directions in hyperspectral data analysis, hyperspectral anomaly detection has emerged as a particularly critical task. Anomalies refer to spectrally distinct pixels that deviate significantly from their local or global background context [20]. Typically modeled as a binary classification problem, anomaly detection aims to distinguish a sparse and rare target class from a more complex and spatially diverse background class as mentioned by [21]. In contrast to classification tasks where the labeling of targets is based on predefined target labels, anomaly detection requires the problem to be handled with limited or no prior knowledge about the characteristics or occurrence of the anomaly, making the task more difficult.

Most of the currently existing anomaly detection algorithms have two basic shortcomings despite their tremendous advances. To begin with they can be very noise-sensitive, especially with high-dimensional hyperspectral data where spectral redundancy and sensor artifacts can impair performance. Second, traditional algorithms rely on the Gaussian distribution of background; however, in practice this assumption is not always valid, particularly when topography, spectral variations or pixel mixture are nonlinear. Indicatively, the popular RX detector is fundamentally based on the proper estimation and inversion of the background covariance matrix, an operation that is ill-posed in high-dimensional environments, particularly when the number of spectral bands is significant compared to the number of available training pixels. Furthermore, traditional algorithms are often unaware of the spatial continuity and low-rank structure of an HSI, and thus are not well suited to cluttered scenes.

In an attempt to address these issues, this paper presents a new hyperspectral anomaly detection system, referred to as L-PCAD, that builds on top of powerful subspace recovery using LADM as a preprocessing step. The process separates the potential anomalies in the sparse representation of the original data and the global structure of the background is kept with the use of low-rank and sparse components by the method. The low-rank background matrix is then obtained and the background matrix forms the basis of the further anomaly detection process.

Moreover, the suggested work suggests some significant improvements to the traditional RX detector in an attempt to achieve accuracy and strength. Instead of using direct inversion of the empirical covariance matrix, which may not be reliable in noisy or ill-conditioned environments, the suggested method regularizes the covariance estimation with a graph Laplacian matrix. This is necessary to facilitate the model to more effectively capture local geometry of data and minimize the effects of noise. After that, the PCA-regularized Laplacian matrix is subjected to principal component analysis (PCA) to identify the most informative spectral features. The two-stage refinement leads to more discriminative noise-resistant anomaly detection especially in complex and heterogeneous hyperspectral scenes.

The summary of the proposed method and the key contributions is as follows:

1. A method is implemented as a preprocessing phase utilizing a LADM-based Robust Subspace Recovery algorithm, effectively separating anomalies from background components and producing a sparse anomaly-relevant representation.
2. A modified RX detector is presented, in which the inverse covariance matrix is replaced with a PCA-regularized Laplacian alternative to improve the preservation of spatial connections in the data.
3. PCA is applied to the Laplacian matrix obtained using the low-rank background matrix, which improves detection performance.

The novelty of the study lies in the construction of the anomaly detector, which is built using a PCA-regularized Laplacian matrix. Unlike previous studies, the Laplacian matrix is computed from the background matrix, rather than the original data. Consequently, the traditional RX detector is enhanced by incorporating both the Laplacian matrix and PCA, resulting in improved detection performance and increased robustness against false alarms.

The remainder of this paper is organized as follows. Section 2 reviews the existing literature on hyperspectral anomaly detection, with a focus on statistical-based, representation-based, and deep learning-based approaches. In Section 3, the details of the proposed method are presented along with a description of the hyperspectral datasets used in the experiments. Section 4 provides experimental results. A detailed analysis and interpretation of the findings are discussed in Section 5. Section 6 presents the conclusion of the current study.

## 2. Related Work

Hyperspectral anomaly detection methods can be broadly categorized into three types: statistical-based, representation-based, and deep learning-based approaches. Statistical methods focus on describing the background using certain probability models to distinguish anomalies from normal patterns. Representation-based techniques, as introduced by [22], take a different approach by aiming to reconstruct the background using a limited dictionary of representative samples. Deep learning has made significant progress in image and speech recognition and has resolved several challenging issues in pattern identification. Therefore, deep features are extracted and analyzed from hyperspectral images to conduct anomaly detection based on deep learning.

### 2.1. Statistical-Based Methods

The most traditional methods of hyperspectral anomaly detection are statistical theory-based [23,24,25]. They use statistical characteristics to detect anomalies and are robust and easy to use. The benchmark method is the Reed–Xiaoli (RX) algorithm proposed in [26]. It relies on a generalized likelihood ratio test. The method assumes the background follows a Gaussian distribution and estimates the corresponding probability density functions by computing the covariance matrix and mean vector from all background samples. It has two primary implementations: the global RX (GRX), which estimates statistics from the entire scene, and the local RX (LRX), which computes statistics from a neighborhood around each pixel [27]. Nevertheless, the standard statistical detectors like RX have many difficulties with complex or nonlinear background distribution. To eliminate such constraints, there has been a rise in the popularity of kernel-based mechanisms [28,29]. They allow identifying minor anomalies that are not separable in the original input space by implicitly projecting data into higher dimensional feature spaces. One of such approaches is the kernel RX (KRX) algorithm [30]. This method captures nonlinear structures within the data. Just like KRX, the collaborative representation detection (CRD) technique can record good detection outcomes in nonlinear settings [31]. It is better especially where the sample size is limited.

### 2.2. Representation-Based Methods

Representation-based methods have been used for hyperspectral anomaly detection. The main idea behind these methods is that the background has low-rank characteristics. Therefore, a background dictionary is constructed to represent normal pixels and identify anomalies as samples that cannot be accurately represented by this dictionary. These methods rely on the principle that hyperspectral data can be effectively decomposed into distinct components that represent the background and anomalies. As explained, the background can be considered low-rank due to its spatial uniformity and spectral similarity, while anomalies appear sparse [25,32,33,34].

Representation-based techniques provide information from both background and anomaly components. This ability gives them the advantage of identifying anomalous pixels [35]. Since anomalous pixels form a small fraction of the entire dataset, they generally present lower probabilities. Therefore, they become ideal for using matrix decomposition methods. In these types of methods, the high-dimensional data is initially decomposed into low-rank and sparse parts using various algorithms. Robust Principal Component Analysis (RPCA) generates low-rank decomposition-based methods in this field [36]. The RPCA-based Go Decomposition (GoDec) algorithm is used to decompose HSI data into low-rank and sparse matrices [37]. These decomposition algorithms have been used in many research studies. Some of them are mentioned and used for evaluation in this study.

The Low-rank and Sparse Matrix Decomposition (LRaSMD) method employs the GoDec algorithm to achieve the decomposition, followed by Euclidean distance calculation on the sparse component to detect anomalies [38]. Another method that uses GoDec is the hyperspectral anomaly detection method based on the Laplacian matrix (HADLAP) [39]. Instead of the Euclidean distance, it measures the Mahalanobis distance to form an anomaly detector. The Robust Subspace Recovery (RoSuRe) algorithm is another method adopted in various studies to improve the accuracy of data decomposition [40,41]. Subsequent improvements incorporated the Mahalanobis distance metric to refine anomaly discrimination within the sparse component [42]. The Sparse and Low-Rank Matrix Decomposition (SLRMD) technique adopts the RoSuRe algorithm for decomposition [43]. Using the extracted sparse matrix, an anomaly detector is built using the Mahalanobis distance. The Hybrid Anomaly Detection Method (HADM) was later developed [44]. Different than previously mentioned methods, the anomaly detector is built using the Laplacian matrix. Integrating this matrix into the detector boosts performance and provides better detection results.

### 2.3. Deep Learning-Based Methods

In the recent years, deep neural networks have been demonstrated to possess grave advantages in terms of their capability to model and generalize intricate data offering a superior capacity to deliver informative characteristics in comparison to traditional approaches. There have been successful applications of deep learning methods in multispectral image classification with good results in many [45,46,47]. However, they are yet to be harnessed adequately in hyperspectral image anomaly detection. The wide use of hyperspectral anomaly detection based on the concept of deep learning, namely on convolutional neural networks (CNNs), is due to its ability to automatically detect the significant characteristics on both spectral and spatial scales [48,49,50]. These methods perform well in detecting anomalies in complicated background scenes [51]. CNN-based techniques usually obtain hyperspectral data cube features that are important and provide anomaly scores directly. The reference data, which consists of externally labeled supervised CNN-based detectors, is usually used to train them and give them a structured learning environment. Nonetheless, the use of labeled anomaly samples narrows down the generalization ability of these models, especially in real-world contexts where anomalies occur in infrequent and unforeseen or previously unknown conditions [52]. Unsupervised and semi-supervised techniques have overcome these limitations. They permit more flexible as well as robust analysis. These hybrid approaches attempt to enhance background modeling and enable detection to be more accurate by utilizing other hyperspectral data [53]. Transfer learning enables the process of adapting to novel scenes with little to no need for a large labeled dataset, which is the issue with HSI annotated anomalies.

## 3. Materials and Methods

This section describes the proposed anomaly detection framework and the hyperspectral datasets used for evaluation. First, an overview of the method is provided, highlighting its key components and the step-by-step process. Then, the real-world hyperspectral datasets as summarized in Table 1 used in the experiments are introduced.

### 3.1. Hyperspectral Datasets

In this section, four publicly available HSI datasets that are used in the experiments are described [54]. The details of the images are given in Table 1. As can be seen from the table, different materials are used as anomalies: fields, planes, and buildings. These materials have different numbers of pixels in total, changing from 42 to 272. They are captured in different locations by the Airborne Visible–Infrared Imaging Spectrometer (AVIRIS) sensor.

**Figure 1:**
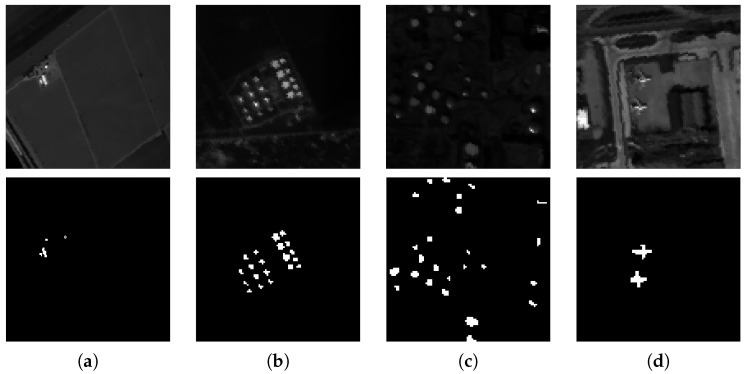
False-color and ground truth images of the: (**a**) Salinas original. (**b**) ABU Urban 2 (**c**) ABU Urban 4. (**d**) ABU Airport 2.

Figure 1 shows the original band images and ground truth images. The first column shows the original band images. The second column presents the anomaly locations. As can be seen from the figure, there are two planes in the last anomaly map. There are many planes in the second anomaly map. In the third one there are many buildings.

### 3.2. Proposed Method

A step-by-step overview of the proposed framework is discussed in this part. The initial phase involves transforming the hyperspectral cube into a matrix representation with a two-dimensional matrix format. Consequently, the hyperspectral image cube $X\in R^{m\times n\times p}$ is represented by a collection of pixel-wise vectors as follows:

(1) $$X=\left[X_{1},X_{2},\ldots ,X_{p}\right],X\in R^{N\times p}$$

where N=m×n denotes the total pixel count, *m* signifies the image height, *n* indicates the image width, and *p* refers to the entire set of spectral channels. Original data can be represented as the addition of background and foreground images as below:

(2) X=B+S

in which *S* is a sparse matrix capturing anomalies, *B* is a low-rank approximation of the background, and *W* denotes a diagonal matrix. The block-diagonal constraint imposed on *W* simplifies the representation by encouraging each band to be reconstructed mainly using related bands within the same block structure, which preserves the intrinsic structure of hyperspectral data. In addition, the constraint $W_{ii}=0$ prevents trivial self-representation, ensuring that each band is reconstructed using information from other bands rather than itself. Equation (2) is transformed into the form

(3) $$X=BW+S,W_{ii}=0$$

Making use of the block-diagonal constraint on *W*, a simple configuration is obtained, in which each band remains unaltered as its own representation. The low-rank representation (LRR) problem [55] is therefore created and represented in Equation (4) below:

(4) $$\begin{array}{cc} \; & \min_{W,S}{∥W∥}_{1}+\lambda {∥S∥}_{1}\\ \; & \;s.t.\;X=BW+S,W_{ii}=0. \end{array}$$

In addressing the problem in Equation (5) using LADM [40,56], the subsequent augmented Lagrangian function is applied:

(5) $$\begin{array}{c} L\left(S,W,Z,\beta \right)={\lambda ∥S∥}_{1}+{∥W∥}_{1}+\langle (X-S)W-(X-S),Z\rangle \;\\ +\frac{\beta }{2}{∥(X-S)W-(X-S)∥}_{F}^{2}. \end{array}$$

In this formulation, *Z* refers to the Lagrange multiplier in the optimization process, and β, being greater than zero, controls the penalty weight. Following the augmented Lagrangian principle, the function L is minimized in parallel for the variables *S* and *W*. To address this issue, ADM solves this problem by breaking down the minimization of L with regard to *S* and *W* into two subproblems as in Equations (6) and (7). $\tilde{W}=I-W$; to update *W* and *S*, the following equations are used:

(6) $$\begin{array}{c} W_{k+1}=\arg\min_{W}{∥W∥}_{1}+\langle B_{k+1}W-B_{k+1},Z_{k}\rangle \;\\ +\frac{\beta }{2}{∥B_{k+1}W-B_{k+1}∥}_{F}^{2} \end{array}$$

(7) $$\begin{array}{c} S_{k+1}=\arg\min_{S}\lambda {∥S∥}_{1}+\langle -B_{k+1}{\tilde{W}}_{k+1},Z_{k}\rangle \;\\ +\frac{\beta }{2}{∥B_{k+1}{\tilde{W}}_{k+1}∥}_{F}^{2} \end{array}$$

The Lagrange multipliers are updated according to the following equation.

(8) $$Z_{k+1}=Z_{k}+\beta_{k}\left(B_{k+1}W_{k+1}-B_{k+1}\right)$$

(9) $$\beta_{k+1}=\rho \beta_{k}$$

After completion of the initial phase of the proposed method, the process continues with the second phase, which involves the formation of the anomaly detector by the Laplace operator. Equation (10) represents the initial form of the RX anomaly detector. Unlike traditional local RX-based methods that rely on sliding windows, the proposed detector in Equation (10) operates globally across the entire scene. By leveraging the low-rank component *B* and the graph-theoretic embedding, the global approach is able to conveniently characterize the entire background distribution and as such minimize the false alarms caused by local spectral variations. The original RX detector is then reformulated by using a PCA-regularized Laplacian matrix. The details of the novel anomaly detector are explained in the following equations.

(10) $$RXD\left(X\right)={\left(X-\mu_{X}\right)}^{T}\Gamma^{-1}\left(X-\mu_{X}\right)$$

where $\mu_{X}$ and $\Gamma^{-1}$ are mean and covariance matrices of *X*, commonly referred to as the precision matrix in the literature. Unlike traditional approaches that compute the covariance matrix directly from the original hyperspectral data, here Γ is derived using a Laplacian representation obtained via PCA, which enhances sensitivity to anomalies. The transition from the standard RX detector in Equation (10) to the final L-PCAD detector is detailed in Equations (11)–(16) where the normalized graph Laplacian is computed, PCA is applied to retain the principal components, and the resulting positive definite covariance matrix *C* is used for inversion.

(11) $$RXD_{L}\left(B\right)={\left(B-\mu_{B}\right)}^{T}L\left(B-\mu_{B}\right)$$

where $\mu_{B}$ denotes the average value computed over the low-rank background matrix, and *L* refers to the graph Laplacian. The *L* matrix is calculated as the difference between the degree matrix *D* and the weight matrix L=D−A. Before using PCA, it is important to emphasize that it is not applied directly to the hyperspectral data. Rather, PCA is used on the Laplacian matrix *L*, which is defined by the low rank background representation *B*. This distinction ensures consistency between the mathematical formulation and the conceptual pipeline, where the covariance structure in the RX detector is replaced by a PCA-regularized Laplacian representation. The weights in *A* are determined via the Cauchy function Equation (12) to emphasize pixels sharing similar features.

(12) $$A_{ij}=\frac{1}{1+{\left(\frac{\mu_{i}-\mu_{j}}{\kappa }\right)}^{2}}$$

where $\mu_{i}$ and $\mu_{j}$ correspond to the mean values of the $i^{th}$ and $j^{th}$ spectral bands. The parameter κ serves as a scaling factor and is computed as the average of all band-wise means.

(13) $$\kappa =\frac{1}{m}\sum_{r=1}^{m}\mu_{r}.$$

Since normalized weights produce improved consistency and analytical benefits, they are employed in the construction of the *L* matrix, which is derived following the formulation in Equation (14).

(14) $$L=D^{-1/2}LD^{-1/2}.$$

Up to now, the original RX detector has been rebuilt by the Laplace matrix. As a final phase, PCA will be adopted in Equation (11). Consider that $\left\{u_{1},u_{2},\ldots ,u_{j}\right\}$ is the set of unit eigenvectors that correspond to $e_{1},e_{2},\ldots ,e_{j}$, the eigenvalues of the covariance matrix L. We can then form the matrix $u=[u_{1}\;u_{2}\;\ldots \;u_{j}]$ with the $i^{th}$ column specified by $u_{i}$. By diagonalizing the matrix *L* into a diagonal matrix *C*, the signal components become decorrelated, since the transformation removes the off-diagonal (correlation) terms. Therefore, PCA is applied to transform the inverse covariance matrix, with the Laplacian matrix acting as a substitute to achieve this decorrelation as expressed by the eigen-decomposition $U^{T}LU=C^{-1}$. Afterwards, dimensionality reduction is performed by projecting the data onto the principal components: $y=U^{T}B$ where typically only the first k<j columns of *U* are used to retain the most significant features. Following the approach mentioned in [57,58], Equation (11) can be rewritten as a function of *y* in the form

(15) $$\begin{array}{ccc} RXD_{L\_PCAD}\left(B\right) & = & B^{T}LB\\ \; & = & {\left(Uy\right)}^{T}L\left(Uy\right)\\ \; & = & y^{T}\left(V^{T}LV\right)y\\ \; & = & y^{T}C^{-1}y\\ \; & = & \sum_{i=1}^{J}e_{i}^{-1}y_{i}^{2} \end{array}$$

where $B=B-\mu_{B}$ and $y_{i}$ represents the *i*th element of the vector y. Since the normalized graph Laplacian *L* represented in Equation (14) is symmetric and positive semi-definite, its eigenvalues are non-negative. During the PCA step, only the components corresponding to non-zero eigenvalues are retained, which yields a diagonal matrix *C* with strictly positive eigenvalues. Therefore, the inverse $C^{-1}$ required in the RX detector is well defined. Given the input image *B*, a likelihood map is generated quantifying the probability that each pixel is anomalous. Consequently, this process results in the creation of anomaly detection maps.

In summary, the method is structured into three sequential phases. First, Equations (6)–(9) are applied to the original data. This step can be called a preprocessing step. Second, *L* is calculated using Equations (12) and (14) with the obtained matrix *B*. A diagonal matrix *C* containing the strictly positive eigenvalues of the normalized Laplacian *L* is obtained, and its inverse $C^{-1}$ is used in the ${RX}_{L-PCAD}$ detector. This ensures that the retained principal components are decorrelated and the anomaly scores are well defined. Finally, the processes mentioned to get Equation (15) are applied to the *L* matrix, and the proposed anomaly detector is built as in Equation (16)

(16) $$RXD_{L\_PCAD}\left(B\right)=y^{T}C^{-1}y$$

Therefore, the final anomaly scores are computed by applying the $RXD_{L\_PCAD}$ detector, where the covariance matrix C is regularized using a graph Laplacian. For clarity, the transition from the standard RX detector to the final L-PCAD detector can be summarized as follows. Starting from the low-rank background matrix *B*, the normalized graph Laplacian

(17) $$L=D^{-1/2}\left(D-A\right)D^{-1/2},$$

is constructed, where *A* is the adjacency matrix (Equation (11)) and *D* is the degree matrix. PCA is applied to *L* to retain only non-zero eigenvalues, producing a positive definite diagonal matrix *C*. The data is then projected onto the principal components,

(18) $$y=U^{T}\left(B-\mu_{B}\right),$$

and the final L-PCAD detector is obtained as

(19) $${\mathrm{RX}}_{L-\mathrm{PCAD}}\left(B\right)=y^{T}C^{-1}y=\sum_{i=1}^{J}e_{i}^{-1}y_{i}^{2}.$$

This concise summary shows how the covariance matrix in the original RX detector is effectively replaced by the PCA-regularized Laplacian for anomaly detection.

## 4. Experimental Results

**Figure 2:**
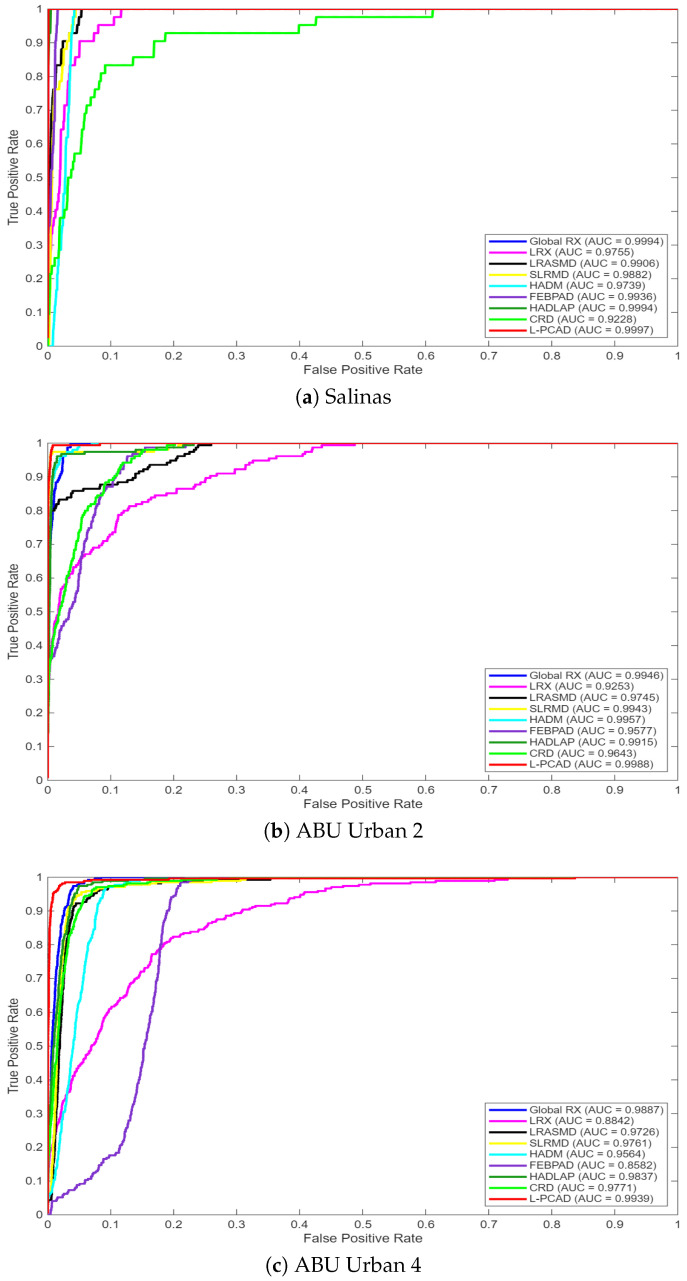

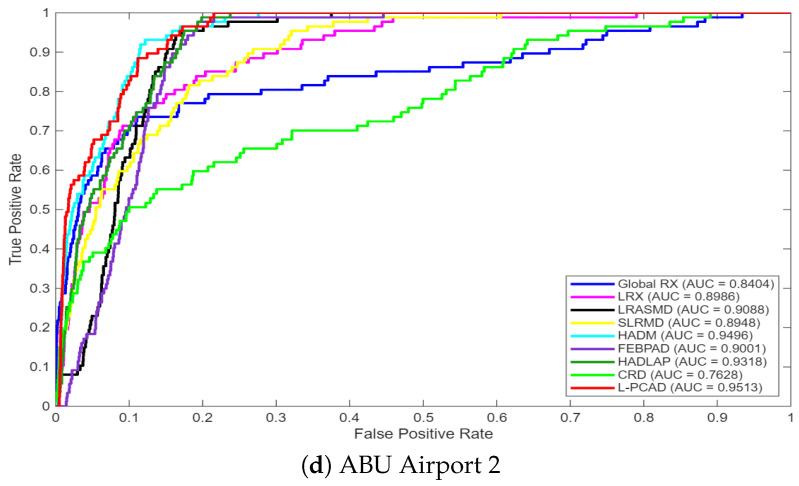
ROC curves of the proposed method (L-PCAD). (**a**) Salinas, (**b**) ABU Urban 2, (**c**) ABU Urban 4, (**d**) ABU Airport 2.

**Figure 3:**
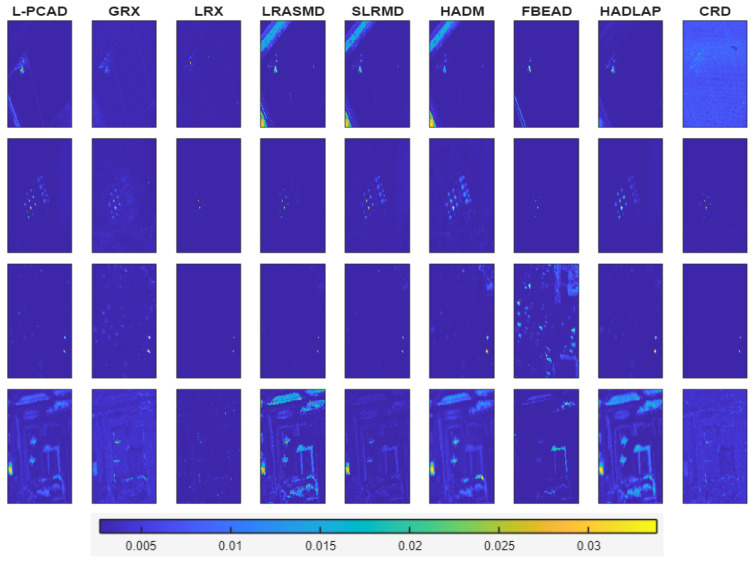
Two-dimensional results for four public HSI datasets for methods.

In this section, the proposed method, L-PCAD, is evaluated in comparison with eight state-of-the-art hyperspectral anomaly detection methods: GRX, LRX, LRASMD, SLRMD, HADM, FEBPAD, HADLAP, and CRD. Experiments are conducted on four HSI datasets. For performance assessment, Receiver Operating Characteristic (ROC) curves are generated and the corresponding area under the curve (AUC) values are computed. Figure 2 presents a comparative evaluation of the anomaly detection methods. The *x*-axis represents the false positive rates, while the *y*-axis indicates true positive rates, also known as the false alarm rate and probability of detection, respectively. AUC values are represented in Table 2. Finally, two-dimensional images showing detected anomaly locations are obtained and presented in Figure 3 to visualize the results.

Figure 2a shows the results of Salinas’ data. As observed, the proposed method, L-PCAD, outperforms all others, achieving a 0.9997 AUC value and higher detection probability at a lower-false alarm rate. It is followed by GRX and HADLAP. Other benchmark methods, such as LRX (0.9755), SLRMD (0.9882), and LRASMD (0.9906), provide acceptable detection accuracy but fall short of the precision offered by the proposed approach. FEBPAD and LRASMD show about 99%; however, they have higher false alarm rates while reaching 100% detection. These results confirm that L-PCAD effectively suppresses background clutter while significantly enhancing target signatures, resulting in the most robust performance across the tested scenarios.

Figure 2b presents the ROC curves for the ABU urban 2 dataset. The proposed method achieves an AUC value of 0.9988, maintaining better performance than all other algorithms. A detailed examination of the ROC curves indicates that L-PCAD reaches a true positive rate (TPR) of approximately 1.0 while the false positive rate (FPR) is still near 0.01, demonstrating exceptional target–background separation in complex urban environments. In contrast, while HADM (0.9957) and Global RX (0.9946) show high overall accuracy, their curves rise less steeply than those of the proposed approach. Other methods such as LRX (0.9253) and FEBPAD (0.9577) exhibit significant performance degradation, requiring a much higher false positive rate, exceeding 0.4 in the case of LRX, to reach full detection capability. These results highlight that L-PCAD not only provides the highest area under the curve but also ensures the lowest possible false alarm rate, which is critical for practical anomaly detection applications.

Figure 2c shows the ROC curves of ABU Urban 4 data. Among all evaluated algorithms, L-PCAD yields the highest AUC value of 0.9939, demonstrating its robust discriminative power. A significant observation from the ROC curves is that while the proposed method achieves a true positive rate of nearly 0.95 at an extremely low FPR of less than 0.05, other competitive methods such as Global RX (0.9887) and HADLAP (0.9837) require higher false alarm rates to reach similar detection levels. Notably, methods like FEBPAD (0.8582) and LRX (0.8842) show a considerably slower ascent, indicating poor background suppression in this specific urban scenario. The rapid increase of the L-PCAD curve, particularly in the lower left part of the plot, confirms its superior ability to extract target signatures from complex backgrounds with minimal false alarms, making it highly suitable for high-precision anomaly detection tasks.

Figure 2d gives the ROC curves of ABU Airport 2 data. In this scenario, L-PCAD achieves the highest AUC value of 0.9513, maintaining its competitive edge even in more challenging detection environments. A quantitative inspection of the curve indicates that the proposed method reaches a TPR of approximately 0.90 at a false positive rate of roughly 0.12, whereas other high-performing methods such as HADM (0.9496) and HADLAP (0.9318) require a wider false alarm range to achieve similar detection sensitivity. In contrast, the performance of CRD (0.7628) and Global RX (0.8404) is notably lower, as their ROC curves exhibit a much slower ascent across the plot. The fact that the L-PCAD curve (red line) consistently remains above all other benchmark curves, especially in the initial stages of the graph, demonstrates its exceptional capability to suppress complex airport background clutter while accurately identifying anomalous targets.

The data in Table 2 is a summary of the AUC values of four hyperspectral image datasets, Salinas, Urban 2, Urban 4 and Airport 2. The numerical scores in every dataset demonstrate that the suggested method yields the most remarkable AUC scores. In particular, the reported method achieves close-to-perfect AUC scores, almost 100% on Salinas data, which is much better than classical algorithms, including GRX and HADLAP. The proposed method has a clear advantage even in more difficult scenarios, like Urban 2, Urban 4 and Airport 2, where the AUCs are 0.9988, 0.9939 and 0.9513, respectively. Other approaches, like GRX, LRX and CRD, on the other hand, do not exhibit any noticeable performance reduction, especially in complex settings as in Airport 2. The results validate the proposed method to achieve better AUC results in various hyperspectral scenes.

Figure 3 illustrates the two-dimensional anomaly maps produced by each method on the four public HSI datasets. The proposed method (L-PCAD) consistently highlights anomalous regions with higher contrast and clearer boundaries compared to competing methods. Traditional RX-based methods (GRX and LRX) often show noisier maps with lower contrast, making anomalies less distinguishable. Low-rank and sparse decomposition methods (LRASMD and SLRMD) improve clarity in some regions but sometimes introduce background artifacts. HADM and FEBPAD detect larger anomalous regions but also highlight false positives in homogeneous areas. HADLAP produces relatively smooth maps but occasionally misses small anomalies, while CRD tends to under-detect subtle anomalous regions. Overall, L-PCAD provides the most visually consistent and precise localization of anomalies across all datasets.

### 4.1. Parameter Setting

The r-adjustment is used to represent a trade-off between suppressing the background signal and preserving anomalous targets, which is often needed in subspace-based hyperspectral detection. Thus, to assist in the configuration of parameters, the rank parameter r was determined empirically for each dataset in order to obtain a balanced detection sensitivity and robustness. Specifically, r = 1 was selected for the Salinas dataset, as it yielded the most stable and consistent performance. Similarly, the Airport 2 dataset also demonstrated optimal results at r = 1, indicating that a lower-rank representation is sufficient to capture the underlying background structure in these scenes. In contrast, the Urban 2 dataset achieved its best performance at r = 2, while the Urban 4 dataset required a slightly higher rank of r = 3, suggesting increased scene complexity and variability. Furthermore, the cardinality parameter k was uniformly defined as k=0.8×M×N for all datasets, ensuring a consistent proportion of selected samples relative to the spatial dimensions of each image. This unified setting enables a fair comparison across datasets while maintaining sufficient representation capacity for anomaly detection. The optimization process employs an adaptive penalty parameter $\beta ={(2∥X∥}_{2}{)}^{-1}$ to ensure scale invariance across different hyperspectral sensors. The convergence rate ρ is set to 1.1, as this gives the best performance results. The regularization parameter λ, which controls the sparsity of the outlier matrix S, is implicitly managed through the soft-thresholding operator. Finally, for the Laplacian and PCA stage, the energy preservation ratio e is set to 1 to maintain the full spectral information. These settings were maintained consistently across all experimental evaluations to verify the autonomous robustness of the proposed method.

**Figure 4:**
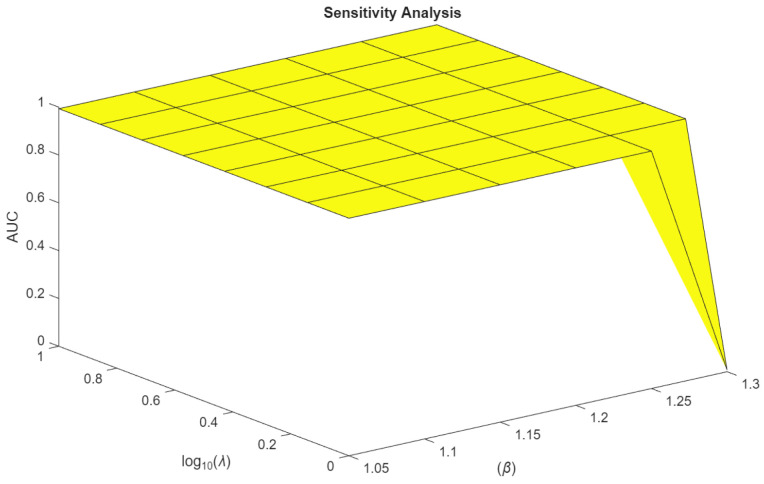
Sensitivity analysis for ABU Urban 4.

The quantitative sensitivity analysis presented in Figure 4 demonstrates the high robustness of the proposed method against variations in hyperparameters, directly addressing the concerns regarding parameter selection. As shown, the AUC performance remains consistently high (above 0.99) across a broad plateau where it $\log_{10}\left(\lambda \right)$ ranges from 0 to 1 and the update rate β varies between 1.05 and 1.25. This optimal region confirms that the algorithm is not hypersensitive to specific tuning and that the adoption of standard empirical conventions (e.g., β=1.1) provides a reliable and stable operational baseline. A significant drop in AUC is only observed at the boundary of β=1.30, where the accelerated growth of the penalty parameter hinders optimal convergence. The numerical results confirm that the system has a high detection accuracy over a large range of configurations, which could then be applied to other hyperspectral data sets without sending a comprehensive calibration set to the device.

### 4.2. Ablation Study

To assess the contribution of each one of the components of the proposed method, a thorough ablation study was performed as presented in Table 3. Only Rosure denotes the use of ROSURE decomposition alone, without applying Laplacian-based processing or PCA. Only L without PCA refers to anomaly detection based solely on the Laplacian matrix, without dimensionality reduction. Only L + PCA indicates that Laplacian-based detection with PCA is applied directly to the original data, without ROSURE decomposition. Rosure + L + PCA + S represents a variant of the proposed framework in which the sparse component (*S*) obtained from ROSURE is used for anomaly detection. Finally, Rosure + L + PCA + B corresponds to the proposed method (L-PCAD), where the background (low-rank) component is utilized together with Laplacian-based PCA for improved anomaly detection. The findings prove that standalone modules such as only Rosure or only L + PCA are a viable baseline in terms of their detection accuracy, but integrating them gives a considerable improvement in detection. One of the essential findings is the results with respect to the ABU Airport 2 dataset; the baseline only L + PCA detector has an AUC of 0.698888, and the suggested Rosure + LAD + B increases the AUC to 0.9513. This significant advancement indicates that the Rosure-based low-rank extraction *B* is an effective way of removing the complicated background noise so that the PCA-regularized Laplacian-based detector (LAD) can isolate the anomalies with far more accuracy. Moreover, the comparison of the proposed method of using the B-based approach with the S-based one (Rosure + L + PCA + S) shows that the low-rank component *B* is generally a more stable and informative input for the detector across all datasets. Finally, the highest AUC scores are observed in all cases when the proposed approach is used which proves that the synergy between the effective subspace recovery and the PCA-regularized Laplacian-based detector (LAD) is the key to the high performance of the anomaly detection.

### 4.3. Computational Complexity Analysis

This section presents the computational complexity analysis of the proposed L-PCAD method in comparison with state-of-the-art baseline anomaly detectors. As shown in Table 4, the proposed L-PCAD method achieves a favorable computational efficiency compared to eight representative baseline methods. Although L-PCAD is based on an iterative optimization framework (LADM), its runtime remains consistently low across all datasets, ranging from 0.0900 to 0.2111 s. In particular, it is comparable to other lightweight methods such as LRASMD and GRX, while significantly outperforming more computationally intensive approaches such as LRX and CRD. The high runtime of LRX (up to 49.23 s on Salinas) highlights the computational burden of covariance inversion in high-dimensional hyperspectral data. In contrast, L-PCAD maintains a stable per-image execution time due to efficient convergence behavior within a limited number of iterations, making it suitable for practical hyperspectral anomaly detection applications.

All experiments were conducted on a workstation with an Intel(R) Core(TM) i7-8750H CPU @ 2.20 GHz, 32 GB RAM, and a 64-bit Windows operating system. The proposed method was implemented in MATLAB R2025b. No GPU acceleration was used in any of the experiments, ensuring a fair comparison among all methods under identical computational conditions.

### 4.4. Validation on Synthetic Data

**Figure 5:**
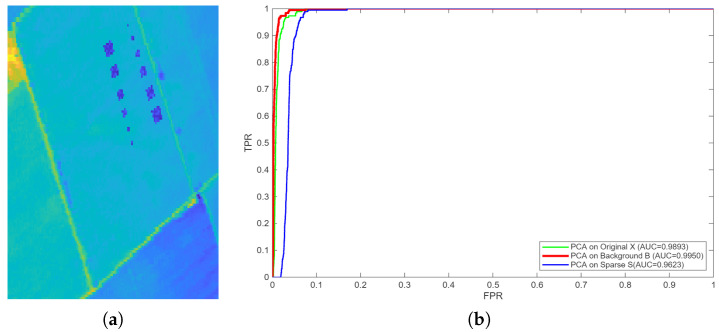

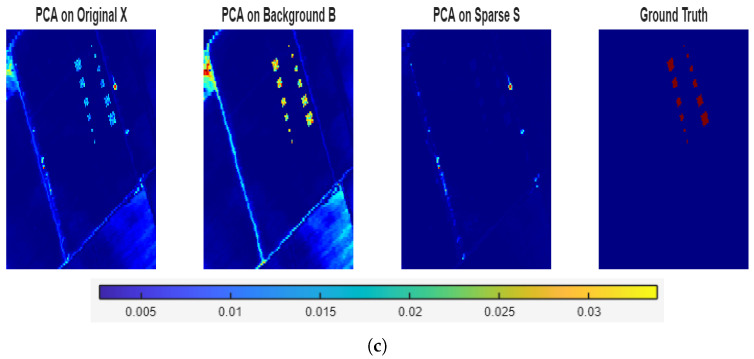
Synthetic image. (**a**) Original band image. (**b**) Roc curve. (**c**) 2D anomaly maps.

The synthetic dataset in Figure 5 used in this study is derived from the Salinas hyperspectral dataset and incorporates artificially implanted anomalies to enable a controlled and interpretable evaluation. The dataset has a spatial size of 126×150 pixels with 204 spectral bands, and contains 185 unreal and artificially implemented anomaly pixels. The implanted anomalies are designed to exhibit varying degrees of spectral similarity to the background, thereby providing both easily distinguishable and more challenging detection scenarios.

Building upon this setup, a toy example is constructed to explicitly illustrate the impact of the proposed Laplacian-based transformation on anomaly separability. In particular, this experiment provides a visual comparison demonstrating that applying PCA on the Laplacian of the low-rank background leads to a more discriminative representation than conventional PCA applied to the original data.

## 5. Discussion

Experimental findings in the form of ROC curves, AUC scores, and two-dimensional anomaly maps can be used to efficiently analyze the functionality of the proposed method in the detection of hyperspectral anomalies. The proposed approach outperforms a number of state-of-the-art methods (GRX, LRX, LRASMD, SLRMD, HADM, FEBPAD, HADLAP, and CRD) in terms of detection on four different datasets. As can be seen from the ROC curves, the proposed technique has increased probabilities of detection throughout the entire range of false alarm rates. Its ROC curve is in most instances much above those of the competing methods and this means that it always dominates. It is important to note that on Salinas the performance of the proposed method is almost optimal in ROC analysis with almost-perfect detection and very few false positives. These results are confirmed by AUC values. The best AUC scores are observed with the proposed method, with a score of 0.9997, 0.9988, 0.9939 and 0.9513 on Salinas, ABU Urban 2, ABU Urban 4, and ABU airport 2 respectively. The experimental data obtained on different datasets show that the proposed approach yields the highest accuracy, though there are cases when the method can be less effective compared to current state-of-the-art approaches such as HADM. Particularly, in ABU Airport 2 data, the improvement is not that significant (0.9988 vs. 0.9957). This can be explained by the saturation of performance; in the cases where a dataset has high spectral contrast and a comparatively simple background structure, most state-of-the-art detectors have reached an AUC value of about 1.0 and have no statistical room to improve. The outcomes of these experiments indicate the high strength of the suggested approach both in organized urban and disorganized natural or airport settings. Moreover, there are two-dimensional anomaly maps generated by the proposed approach that are consistent with the quantitative indicators. These maps are visual confirmation that the method is effective in terms of localizing the anomalies with a high level of specificity in space and low background noise. On the other hand, the baseline methods are prone to either piece or noisy detection especially in a cluttered environment. Overall, the high scores for AUC, the consistently high ROC performance and the visual accuracy of anomaly localization in the anomaly maps are strong indicators that the devised method has substantial differences in hyperspectral anomaly detection. It is highly promising to apply it in real-world remote sensing applications because it is strong in a variety of scenes and conditions.

The experimental results should be interpreted within the context of the anomaly ratios provided in Table 1, as the proposed L-PCAD method demonstrates exceptional sensitivity to rare spectral signatures in highly imbalanced scenarios. For the Salinas and ABU Airport 2 datasets, where anomalous pixels constitute only 0.14% and 0.87% of the total scene respectively, the background dominates over 99% of the data; thus, even a marginal point increase in AUC (e.g., the 0.0017 improvement in Airport 2) represents a statistically non-trivial advancement in detection precision. This refined background suppression capability is particularly evident in the lower false positive rate regions of the ROC curves, highlighting the model’s effectiveness in isolating targets from vast background clutter. Furthermore, the method maintains its robustness on the ABU Urban 2 dataset despite a relatively low anomaly ratio of 1.55%, successfully capturing targets with high confidence. Even in the more complex ABU Urban 4 scene, where the anomaly ratio increases to 2.72%, L-PCAD consistently outperforms benchmark methods, confirming its superior discriminative power across varying levels of target sparsity.

The ablation experiment proves that the high effectiveness of the offered method is not mere chance but rather the result of the synergistic interaction of its main modules. Although the standalone only LAD detector is a good baseline, its work is usually impaired by intricate background clutter as was the case in the ABU Airport 2 results. Using Rosure to extract the low-rank component first, before PCA, removes the noise and outliers, which effectively smooths the background of the data, and so it makes sense that the Cauchy-distance PCA operates with a much higher AUC, between 0.69888 and 0.9513. An important result of our ablation experiment is the comparison of the Rosure + LAD + B and the Rosure + LAD + S setups. Despite the fact that the sparse matrix S is full of outliers, application with the recovered low-rank matrix B as the input of LAD-PCA gives more stable and better AUC values (e.g., 0.9997 in Salinas). This implies that the low-rank element still carries the background structure of the background vital information and the detector can better detect anomalies as statistical deviations of this highly refined subspace.

Despite the strong performance demonstrated in the experimental evaluation, the proposed method has some limitations. First, the experimental evaluation is limited to four publicly available AVIRIS datasets, as additional hyperspectral cameras or datasets for acquiring new data are not available. Therefore, the generalizability of the method to hyperspectral data from other sensors remains to be investigated. Second, although the method shows robust detection in complex scenes, its improvement over state-of-the-art methods may be limited on datasets with very high spectral contrast and relatively simple background structures, as observed in ABU Airport 2. In such cases, the performance of most detectors, including the proposed one, is close to optimal, leaving limited room for further enhancement. Finally, the computational cost associated with low-rank extraction and PCA-based Laplacian construction may be significant for very large datasets, which should be considered in real-world applications.

These limitations provide guidance for future work, including testing on additional datasets from different sensors, exploring adaptive strategies for varying background complexities, and optimizing computational efficiency for large-scale hyperspectral data.

## 6. Conclusions

An anomaly detection method is suggested in this research for application to hyperspectral images. It is assessed on four benchmark datasets. A plethora of experiments prove that the proposed method has better performance than many existing ones, such as GRX, LRX, LRASMD, SLRMD, HADM, FEBPAD, HADLAP, and CRD. Quantitative measurements derived using ROC curves and values of AUC indicate that the proposed method outperformed all the other results in terms of the highest or close-to-highest scores of AUC in all cases. In addition, the numerical findings were complemented by the qualitative results in the form of two-dimensional anomaly maps. The proposed approach is able to visualize high-spatial-precision anomalies with minimal background noise and is able to localize anomalies with high spatial accuracy and clarity, which provides superior visual interpretability compared to other methods. Summarizing the points discussed above, the suggested approach has a high detection rate and precision in a variety of hyperspectral conditions. The features of these attributes render it a hopeful solution for practical applications in the real world like environmental monitoring, defense, and remote sensing analytics.

## Figures and Tables

**Table 1:** Hyperspectral dataset details.

Attribute	Salinas	ABU Urban 2	ABU Urban 4	ABU Airport 2
Spatial Size	150 × 200	100 × 100	100 × 100	100 × 100
Bands	204	207	205	205
Resolution	3.7 m	7.1 m	7.1 m	7.1 m
Location	Salinas Valley	Los Angeles	Los Angeles	Los Angeles
Sensor	AVIRIS	AVIRIS	AVIRIS	AVIRIS
Anomaly Type	Field	Plane	Buildings	Plane
Anomaly Pixels	42	155	272	87

**Table 2:** AUC values for each method on different HSI datasets.

Method	Salinas	ABU Urban 2	ABU Urban 4	ABU Airport 2
Proposed method	0.9997	0.9988	0.9939	0.9513
GRX	0.9994	0.9946	0.9887	0.8404
LRX	0.9755	0.9253	0.8842	0.8986
LRASMD	0.9906	0.9745	0.9726	0.9088
SLRMD	0.9882	0.9943	0.9761	0.8948
HADM	0.9739	0.9957	0.9564	0.9496
FEBPAD	0.9936	0.9577	0.8582	0.9001
HADLAP	0.9994	0.9915	0.9837	0.9318
CRD	0.9228	0.9643	0.9771	0.7628

**Table 3:** AUC values of ablation study.

Method	Salinas	ABU Urban 2	ABU Urban 4	ABU Airport 2
only Rosure	0.98339	0.99523	0.96511	0.67393
only L without PCA	0.99721	0.99561	0.99249	0.76175
only L + PCA	0.99815	0.99811	0.99352	0.69888
Rosure + L + PCA + S	0.97681	0.9969	0.96438	0.93309
Rosure + L + PCA + B (proposed)	0.9997	0.9988	0.9939	0.9513

**Table 4:** Runtime (seconds) for each method on different HSI datasets.

Method	Salinas	ABU Urban 2	ABU Urban 4	ABU Airport 2
Proposed method (L-PCAD)	0.2111	0.0900	0.0989	0.1175
GRX	0.3540	0.1338	0.1292	0.1398
LRX	49.2340	14.5261	13.6912	15.9754
LRASMD	0.0927	0.0308	0.0292	0.0386
SLRMD	0.3357	0.1372	0.1205	0.1351
HADM	6.9048	2.2486	2.2026	2.7653
FEBPAD	10.4591	2.8186	2.7943	2.9695
HADLAP	6.9547	2.1964	1.9271	2.6911
CRD	29.8081	9.7986	9.0589	12.3349

## Data Availability

The original data presented in the study are openly available in the Salinas dataset at https://ieee-dataport.org/documents/hyperspectral-remote-sensing-scenes (accessed on 3 February 2026) and in the ABU dataset at https://xudongkang.weebly.com/data-sets.html (accessed on 3 February 2026).
